# Impact of Elevated AMH Levels on Maternal and Perinatal Outcomes in IVF Pregnancies with PCOS

**DOI:** 10.3390/jcm14196706

**Published:** 2025-09-23

**Authors:** Ayse Cigdem Bayrak, Recep Taha Ağaoğlu, Berna Seyhan, Zehra Vural Yılmaz

**Affiliations:** 1 Department of Perinatology, Ankara Etlik City Hospital, Ankara 06170, Turkey; drcigdembayrak@gmail.com (A.C.B.); zehravural@gmail.com (Z.V.Y.); 2 Department of Obstetrics and Gynecology, Ankara Etlik City Hospital, Ankara 06170, Turkey; drb3rna@gmail.com

**Keywords:** anti-Müllerian hormone, in vitro fertilization, polycystic ovary syndrome, perinatal outcome

## Abstract

**Objective:** To investigate whether polycystic ovary syndrome (PCOS) is associated with increased maternal and perinatal complications in in vitro fertilization (IVF) pregnancies, and to evaluate the relationship between anti-Müllerian Hormone (AMH) levels and adverse maternal and perinatal outcomes within the PCOS group. **Methods:** This retrospective cohort included 424 women with singleton IVF pregnancies delivered at Ankara Etlik City Hospital between September 2022 and June 2025. Participants were classified as PCOS (n = 106; AMH ≥ 4.5 ng/mL) or non-PCOS (n = 318; AMH 1.0–4.5 ng/mL). Maternal outcomes were gestational diabetes mellitus (GDM) and preeclampsia, while perinatal outcomes included preterm birth, small-for-gestational-age (SGA), large-for-gestational-age (LGA), 5-min Apgar ≤ 7, and neonatal intensive care unit (NICU) admission. Composite adverse maternal outcomes (CAMO) and composite adverse perinatal outcomes (CAPO) were defined as the occurrence of at least one respective complication. Outcomes were compared between groups, and multivariable logistic regression identified predictors of CAMO and CAPO in the PCOS cohort. **Results:** Women with PCOS had significantly higher incidences of GDM and preeclampsia compared to controls (*p* < 0.05 for all). CAMO was more common in the PCOS group (34.0% vs. 11.9%, *p* < 0.001). Median gestational age at delivery was lower among women with PCOS (*p* = 0.026). Rates of LGA neonates, low 5-min Apgar scores, and NICU admissions were significantly higher in the PCOS group (*p* < 0.001 for each). CAPO rates were comparable between groups (*p* = 0.132). In multivariable models, AMH level remained an independent predictor of both CAMO and CAPO (*p* = 0.002 and *p* = 0.014, respectively). **Conclusions:** Women with PCOS and elevated preconception AMH levels are at increased risk for both maternal metabolic complications and adverse neonatal outcomes following IVF conception. These findings suggest that preconception AMH levels, when interpreted alongside a PCOS diagnosis, may help identify women at higher obstetric and perinatal risk.

## 1. Introduction

Physiological aging of the ovaries is an inevitable process; however, when this decline occurs prematurely or progresses at an accelerated rate, it becomes a major underlying cause of reproductive disorders. Beyond their essential roles in folliculogenesis and ovulation, the ovaries are central regulators of multiple endocrine pathways that influence overall reproductive health [1,2].

In recent years, the increasing prevalence of advanced maternal age has heightened interest in the evaluation of ovarian reserve [3]. Although substantial advances have been made in understanding ovarian pathophysiology, accurately estimating ovarian age with high precision remains challenging [4]. One of the most widely used biomarkers for this purpose is anti-Müllerian hormone (AMH). AMH is considered one of the most informative markers for assessing ovarian reserve and is now a routine component of clinical evaluation. It provides a practical estimate of the remaining follicular pool, offering valuable guidance for fertility counseling and treatment planning [5].

Lower AMH levels are associated with a reduced response to ovarian stimulation and an increased likelihood of premature ovarian insufficiency [6]. In contrast, AMH concentrations are often significantly elevated in women with polycystic ovary syndrome (PCOS). This increase is primarily attributed to the greater number of small antral follicles, which are the main source of AMH [7,8]. PCOS is a complex reproductive disorder characterized by oligo-anovulation, hyperandrogenism, and polycystic ovarian morphology. It is the most common endocrine disorder, affecting approximately 5–20% of women of reproductive age, and is associated with substantial metabolic consequences [9]. Among these, insulin resistance is both the most prevalent and the most clinically relevant metabolic abnormality [10].

Alterations in AMH levels have been linked to metabolic disturbances across multiple studies. In pregnant women, Zhang et al. reported that higher AMH concentrations were associated with an increased risk of gestational diabetes, whereas Güler et al. demonstrated that lower AMH levels were associated with a higher risk of cardiovascular disease [11,12]. In women with PCOS, elevated AMH levels have also been associated with an increased risk of pregnancy complications, suggesting that this biomarker may reflect broader metabolic and obstetric risks [13]. In light of these considerations, the present study aimed to investigate whether the presence of PCOS is associated with an increased risk of maternal and perinatal complications in pregnancies conceived via IVF.

## 2. Materials and Methods

This retrospective cohort study was conducted at a tertiary maternity hospital between September 2022 and June 2025. The ethics committee of our center approved the study protocol (Protocol ID: AEŞH-BADEK2-2025-386), and the study was conducted in accordance with the universal ethical standards of the Declaration of Helsinki, waiving informed consent due to the retrospective nature of the study.

We reviewed the electronic medical records of all women with IVF-conceived pregnancies who delivered at our institution during the study period. The study population was divided into two groups according to the presence or absence of PCOS.

The PCOS group comprised women diagnosed with PCOS by infertility specialists prior to IVF, based on clinical and/or biochemical features consistent with the Rotterdam criteria. Although specific PCOS phenotypes (e.g., anovulatory, hyperandrogenic, or combined) were not documented in the medical records, to reduce heterogeneity and specifically investigate the impact of elevated AMH, we restricted this group to women with PCOS who also had an AMH level ≥ 4.5 ng/mL measured within one year prior to conception. In our dataset, the cut-off was applied to create a more homogeneous study group and to examine the relationship between AMH levels and perinatal outcomes.

The control group consisted of women without PCOS who underwent IVF for other indications and had AMH levels between 1.0 and 4.5 ng/mL measured within the same timeframe. Women with diminished ovarian reserve were excluded to avoid confounding effects of advanced maternal age and low ovarian reserve on pregnancy outcomes, and to better represent the general IVF population, the comparison group was randomly selected and matched to the PCOS group in a 1:3 ratio based on maternal age.

Patients were excluded if they had pregestational diabetes mellitus or impaired glucose tolerance diagnosed at the initial antenatal visit, a known history of gestational diabetes mellitus (GDM) in previous pregnancies, or a history of ovarian surgery. Additional exclusion criteria included the presence of comorbidities other than PCOS, as well as the use of corticosteroids or hormonal medications before pregnancy.

Clinical and demographic data, including maternal age, gravidity, parity, history of abortion, height, pre-pregnancy weight, and underlying cause of infertility, were obtained from electronic medical records. Antenatal follow-up records were reviewed to assess oral glucose tolerance test (OGTT) results, confirm the diagnosis of GDM, and classify its management as either diet-controlled (GDM A1) or requiring pharmacologic intervention (GDM A2). Other pregnancy-related comorbidities, such as preeclampsia, were also recorded. Composite adverse maternal outcome (CAMO) was defined as the presence of any of the following conditions during pregnancy: GDM A1, GDM A2, or preeclampsia.

Perinatal outcomes included mode of delivery, gestational age at birth (weeks), preterm birth (<37 weeks), birth weight, small for gestational age (SGA; <10th percentile) and large for gestational age (LGA; >90th percentile) according to the INTERGROWTH-21st chart, 5-min Apgar score ≤ 7, and neonatal intensive care unit (NICU) admission. To minimize potential bias and confounding, we excluded multiple pregnancies, and fetuses with structural or genetic anomalies.

All statistical analyses were conducted utilizing IBM SPSS Statistics for Windows, Version 27.0 (Armonk, NY, USA: IBM Corp., 2015). The distribution of continuous variables was evaluated using both visual methods (histograms and probability maps) and statistical methods (Kolmogorov–Smirnov and Shapiro–Wilk tests). For normally distributed data, findings were presented as mean ± standard deviation (SD), and group comparisons were conducted using the independent samples *t*-test. For non-normally distributed data, values were expressed as median and interquartile range (IQR), with comparisons conducted using the Mann–Whitney U test. Categorical variables were examined via the Chi-square test. Correlation analyses were performed according to variable types and distributions. Pearson’s correlation test was utilized to evaluate correlations between two normally distributed continuous variables, whereas Spearman’s rank correlation test was employed for non-normally distributed continuous variables. Point-biserial correlation analysis was employed to examine the relationship between a continuous variable and a dichotomous variable. Univariable and multivariable logistic regression analyses were conducted to assess the independent associations of various clinical characteristics with composite adverse maternal outcomes (CAMO) and composite adverse perinatal outcomes (CAPO) in the PCOS cohort. The strength of relationships was expressed as odds ratios (OR) and adjusted odds ratios (aOR), accompanied by 95% confidence intervals (CI). A two-tailed *p*-value of less than 0.05 was considered statistically significant.

## 3. Results

### 3.1. Baseline Characteristics

A total of 424 participants were included in the study, comprising 106 women with PCOS and 318 women without PCOS. All women in the PCOS group had AMH levels ≥ 4.5 ng/mL, while the non-PCOS group included only those with AMH levels between 1.0 and 4.5 ng/mL. There were no significant differences between the groups in terms of maternal age, gravidity, parity, or history of abortion (*p* > 0.05). The mean pre-pregnancy BMI was significantly higher in the PCOS group (25.2 [IQR: 22.21–28.91]) compared to the non-PCOS group (23.78 [IQR: 21.30–26.67]; *p* = 0.002). Additionally, the proportion of women with BMI ≥ 30 kg/m^2^ was greater in the PCOS group (21.7% vs. 7.5%).

In the non-PCOS group, infertility etiologies included male factor infertility, tubal factor, and unexplained infertility. The distribution of the infertility etiologies, along with other clinical and demographic characteristics, is presented in Table 1.

### 3.2. Maternal Outcomes

The incidence of GDM A1, GDM A2, total GDM, and preeclampsia was significantly higher in the PCOS group compared to the non-PCOS group (*p*-values: 0.021, <0.001, <0.001, and 0.016, respectively). The frequency of composite adverse maternal outcomes (CAMO) was also significantly elevated in the PCOS group (34.0%) compared to the control group (11.9%) (*p* < 0.001) (Table 2).

### 3.3. Perinatal Outcomes

The median gestational age at birth was significantly lower in the PCOS group compared to the non-PCOS group (38 [37–39] weeks vs. 39 [38–39] weeks; *p* = 0.026). Birth weights were comparable between groups (*p* = 0.423), and no significant difference was observed in preterm birth rates (14.2% vs. 15.4%; *p* = 0.754). The incidence of LGA was significantly higher in the PCOS group (13.2% vs. 4.1%; *p* < 0.001), while the incidence of SGA was greater in the non-PCOS group (15.1% vs. 7.5%; *p* = 0.046). Additionally, the rates of 5-min Apgar scores ≤ 7 (10.4% vs. 2.5%; *p* < 0.001) and NICU admission (15.1% vs. 2.5%; *p* < 0.001) were significantly higher in the PCOS group. Although the incidence of composite adverse perinatal outcomes (CAPO) was higher in the PCOS group (43.4%) than in the non-PCOS group (35.2%), this difference did not reach statistical significance (*p* = 0.132) (Table 2).

### 3.4. Subgroup Analyses Within the PCOS Group

Among women in the PCOS cohort, AMH levels were significantly higher in those who developed CAMO compared to those who did not (*p* = 0.008), while no significant difference in AMH levels was observed between those with and without CAPO (*p* = 0.302) (Table 3).

Correlation analysis revealed weak positive correlations between AMH levels and birth weight (r = 0.267, *p* = 0.006), gestational hypertensive disorders (r = 0.292, *p* = 0.002), NICU admission (r = 0.325, *p* < 0.001), and CAOO (r = 0.259, *p* = 0.007). A weak negative correlation was found with preterm birth (r = −0.233, *p* = 0.016), while no significant correlation was found between AMH levels and CAPO (r = 0.101, *p* = 0.304) (Table 4).

In the univariate logistic regression analysis, both pregestational BMI (OR: 1.144; 95% CI: 1.044–1.254; *p* = 0.004) and AMH level (OR: 1.213; 95% CI: 1.071–1.373; *p* = 0.002) were significantly associated with CAMO. In the multivariate model adjusted for maternal age and BMI, AMH remained an independent predictor of CAMO (aOR: 1.221; 95% CI: 1.075–1.387; *p* = 0.002).

Regarding CAPO, univariate analysis identified only gestational age at birth as a significant factor (OR: 0.615; 95% CI: 0.459–0.824; *p* = 0.001), while AMH level, maternal age, and BMI were not significant predictors (Table 5). However, in the multivariate model adjusting for gestational age and maternal age, AMH level was found to be independently associated with CAPO (aOR: 1.173; 95% CI: 1.032–1.332; *p* = 0.014).

## 4. Discussion

PCOS is widely recognized as a common endocrine disorder characterized by chronic anovulation, hyperandrogenism, and polycystic ovarian morphology. Elevated AMH levels are frequently observed in women with PCOS and are primarily attributed to an increased number of small antral follicles and altered granulosa cell function. This hormonal profile reflects not only disrupted folliculogenesis but also underlying endocrine abnormalities, including excessive androgen production [14]. Although various age-specific AMH thresholds have been proposed in the literature, levels above 4.5 ng/mL are consistently reported in women with PCOS [15], and they are typically higher in the classic phenotypes of the disorder [16,17]. In our retrospective dataset, this threshold was applied to define a homogeneous study group without other coexisting medical conditions. Building on this context, we aimed to investigate the association between elevated AMH levels and perinatal outcomes in women with PCOS.

Building on this physiological and metabolic context, our study aimed to evaluate whether the presence of PCOS—a condition characterized by elevated AMH levels—is associated with increased maternal metabolic complications and adverse neonatal outcomes in IVF pregnancies. By comparing outcomes between women with and without PCOS, we sought to clarify the gestational risks linked to this common endocrine disorder.

Numerous studies have examined the impact of PCOS on maternal and perinatal outcomes. A systematic review and meta-analysis by Yu et al. demonstrated that a preconception diagnosis of PCOS is associated with an increased risk of adverse pregnancy, fetal, and neonatal outcomes [18]. Similarly, Aktun et al. reported a higher incidence of GDM in women with PCOS [19]. In our study, women with PCOS exhibited higher rates of both maternal and perinatal adverse outcomes compared to those without PCOS, and these associations remained significant after adjusting for obesity. This finding is consistent with the study by Mustaniemi et al., which suggested that the elevated risk of GDM in women with PCOS may be largely influenced by coexisting factors such as advanced maternal age and increased BMI [20].

Our findings also highlight potential fetal consequences of PCOS, including higher rates of LGA neonates, NICU admissions, and low 5-min Apgar scores. These outcomes may reflect the impact of PCOS-related metabolic disturbances on placental function and fetal development. Similar associations have been reported by Boomsma et al. [21] and Palomba et al. [22], who observed higher rates of macrosomia and adverse neonatal outcomes in pregnancies affected by PCOS. In contrast, the control group had a higher incidence of SGA neonates, which may be explained by the absence of the hyperinsulinemic and hyperandrogenic metabolic environment that can promote fetal overgrowth.

Despite AMH levels being an independent predictor of unfavorable maternal and perinatal outcomes in our multivariable models, the effect sizes were modest. In our subgroup analysis, elevated AMH levels were independently associated with CAMO (aOR 1.22) and CAPO (aOR 1.17), even after adjusting for maternal age and BMI. While maternal age was not an independent predictor, BMI emerged as a significant factor, consistent with its established role in pregnancy complications. Although these associations reached statistical significance, the effect sizes were modest and close to unity, suggesting limited value for individual risk prediction. Nonetheless, AMH may still have clinical relevance when interpreted together with maternal age and BMI, contributing to a multifactorial risk profile rather than serving as a strong independent predictor. For perinatal outcomes, maternal age and BMI were not significant, whereas gestational age at delivery was the dominant determinant.

The mechanisms are likely multifactorial, including insulin resistance, inflammation, endothelial dysfunction, and altered placental angiogenesis [23,24]. These changes may impair placental development, increasing the risk of hypertensive disorders and preterm birth. Our findings support closer prenatal surveillance in women with PCOS, even without obesity or overt metabolic disease. Although AMH may help identify high-risk cases, its predictive value for obstetric outcomes requires confirmation in prospective studies. Preventive strategies remain crucial, including preconception weight optimization and lifestyle modification, which are central to reducing maternal and perinatal risks [25,26]. In this regard, elevated AMH might serve as an early signal to consider timely preventive measures, such as preeclampsia prophylaxis with low-dose aspirin in appropriate candidates or earlier screening for gestational diabetes in women with additional risk factors. These approaches underscore the need for individualized management of women with PCOS.

A major strength of this study is the use of a clearly defined IVF-conceived singleton cohort, which allowed for standardized documentation of infertility diagnoses, AMH levels, and pregnancy outcomes. Excluding women with diminished ovarian reserve from the control group minimized confounding, while multivariate analyses accounted for key factors such as maternal age and BMI. However, several limitations should be noted. The retrospective design may have introduced selection and information bias. The lack of detailed hormonal, metabolic, and ultrasonographic data limited further characterization of PCOS severity. Additionally, as all participants conceived via IVF, these findings may not be generalizable to spontaneous pregnancies.

## 5. Conclusions

In our cohort, women with PCOS had an increased risk of both maternal metabolic complications and adverse perinatal outcomes. These findings suggest that the endocrine and metabolic disturbances associated with PCOS contribute significantly to these risks, and that high AMH levels may help identify women at greater risk. While AMH may reflect underlying metabolic dysregulation, its predictive value for obstetric outcomes remains to be confirmed. Our results emphasize the importance of individualized management strategies in pregnancies complicated by reproductive disorders such as PCOS. However, validation in larger prospective studies is needed to better define these associations and their impact on perinatal outcomes.

## Figures and Tables

**Table 1:** Comparison of maternal clinical and demographic characteristics between groups.

Variables	PCOS Group (n = 106)	Control Group (n = 318)	*p* Value
Maternal age (years)	30 (27–33)	30 (27–34)	0.774 ^1^
Gravidity	1 (1–2)	1 (1–2)	0.749 ^1^
Parity	0 (0–0)	0 (0–0)	0.076 ^1^
Abortus	0 (0–0)	0 (0–0)	0.648 ^1^
Pre-pregnancy BMI (kg/m^2^)	25.27 (22.21–28.91)	23.78 (21.30–26.67)	0.002 ^1^
Obesity (BMI ≥ 30 kg/m^2^)	23 (21.7%)	24 (7.5%)	<0.001 ^2^
Cause of infertility			
Normogonadotropic anovulation (WHO Group II) *	106 (100%)	—	—
Unexplained infertility	—	130 (40.8%)	—
Male factor	—	126 (39.6%)	—
Tubal factor	—	42 (13.2%)	—
Combined factors	—	12 (3.7%)	—
Others	—	8 (2.5%)	—

^1^ The Mann–Whitney U test was used for comparisons between groups. Data are presented as median (interquartile range). ^2^ Categorical variables were compared using the chi-square or Fisher’s exact test, as appropriate. Results are shown as n (%). *: WHO Group II (normogonadotropic anovulation) includes only patients diagnosed with. Abbreviation: PCOS; Polycystic Ovary Syndrome; BMI, Body Mass Index; WHO, World Health Organization.

**Table 2:** Comparison of maternal and perinatal outcomes between the PCOS and the Control Group.

Variables	PCOS Group (n = 106)	Control Group (n = 318)	*p*-Value
**Maternal complications**			
GDM A1	16 (15.1%)	24 (7.5%)	0.021 ^1^
GDM A2	16 (15.1%)	7 (2.2%)	<0.001 ^1^
Overall GDM	32 (30.2%)	31 (9.7%)	<0.001 ^1^
Preeclampsia	8 (7.5%)	7 (2.2%)	0.016 ^1^
CAMO	36 (34.0%)	38 (11.9%)	<0.001 ^1^
Route of delivery			
Vaginal delivery	42 (39.6%)	145 (45.6%)	
Cesarean section	64 (60.4%)	173 (54.4%)	
**Perinatal outcomes**			
Gestational age at birth	38 (37–39)	39 (38–39)	0.026 ^1^
Birth weight (g)	3190 (2850–3400)	3130 (2750–3440)	0.423 ^2^
Preterm birth (<37 weeks)	15 (14.2%)	49 (15.4%)	0.754 ^2^
SGA (<10th percentile)	8 (7.5%)	48 (15.1%)	0.046 ^1^
LGA (>90th percentile)	14 (13.2%)	13 (4.1%)	<0.001 ^1^
5-min Apgar score ≤ 7	11 (10.4%)	8 (2.5%)	<0.001 ^1^
NICU admission	16 (15.1%)	8 (2.5%)	<0.001 ^1^
CAPO	46 (43.4%)	112 (35.2%)	0.132 ^1^

^1^ Categorical variables were compared using the chi-square or Fisher’s exact test, as appropriate. Results are shown as n (%). ^2^ The Mann–Whitney U test was used for comparisons between groups. Data are presented as median (interquartile range). Abbreviation: PCOS, Polycystic Ovary Syndrome; GDM, Gestational Diabetes Mellitus; CAMO, Composite Adverse Maternal Outcome; SGA, Small for Gestational Age; LGA, Large for Gestational Age; NICU, Neonatal Intensive Care Unit; CAPO, Composite Adverse Perinatal Outcome.

**Table 3:** Comparison of AMH Levels Between CAMO and CAPO Subgroups in the PCOS Population.

Subgroup		AMH (Median, IQR)	*p*-Value
CAMO	CAMO	9.00 (5.93–12.79)	0.008 ^1^
	Non-CAMO	6.46 (5.08–7.93)	
CAPO	CAPO	7.27 (5.41–9.41)	0.302 ^1^
	Non-CAPO	6.55 (5.07–8.77)	

^1^ The Mann–Whitney U test was used for comparisons between groups. Data are presented as median (interquartile range). Abbreviation: AMH, Anti-Müllerian Hormone; PCOS, Polycystic Ovary Syndrome; CAMO, Composite Adverse Maternal Outcome; CAPO, Composite Adverse Perinatal Outcome.

**Table 4:** Correlation Between Anti-Müllerian Hormone (AMH) Levels and Obstetric/Perinatal Outcomes in the PCOS Group.

Variables	r	*p*-Value
Gestational Age at Birth (weeks)	0.173	0.076 ^1^
Birth Weight (g)	0.267	0.006 ^1^
GDM A1	0.053	0.590 ^2^
GDM A2	0.146	0.135 ^2^
Overall GDM	0.155	0.112 ^2^
Preeclampsia	0.292	0.002 ^2^
SGA	0.044	0.656 ^2^
LGA	0.122	0.214 ^2^
NICU Admission	0.325	<0.001 ^2^
Preterm Birth	−0.233	0.016 ^2^
CAMO	0.259	0.007 ^2^
CAPO	0.101	0.304 ^2^

^1^ The correlations between two continuous variables were assessed using Spearman’s rank correlation analysis. ^2^ The correlations between continuous and dichotomous (binary categorical) variables were assessed using Point-biserial correlation analysis. Abbreviation: PCOS, Polycystic Ovary Syndrome; GDM, Gestational Diabetes Mellitus; CAMO, Composite Adverse Maternal Outcome; SGA, Small for Gestational Age; LGA, Large for Gestational Age; NICU, Neonatal Intensive Care Unit; CAPO, Composite Adverse Perinatal Outcome.

**Table 5:** Logistic regression analyses for the association between AMH levels and composite adverse outcomes in the PCOS Group.

Variables	Univariable		Multivariable	
	OR (95% CI)	*p* Value	aOR (95% CI)	*p* Value
**For CAMO**				
Maternal age (years)	0.992 (0.902–1.091)	0.867	0.996 (0.891–1.113)	0.937
BMI (kg/m^2^)	1.144 (1.044–1.254)	0.004	1.156 (1.048–1.275)	0.004
AMH	1.213 (1.071–1.373)	0.002	1.221 (1.075–1.387)	0.002
**For CAPO**				
Maternal age (years)	1.078 (0.981–1.185)	0.117	1.096 (0.987–1.218)	0.087
BMI (kg/m^2^)	0.989 (0.910–1.075)	0.793	0.970 (0.884–1.065)	0.527
Gestational age at birth (weeks)	0.615 (0.459–0.824)	0.001	0.537 (0.383–0.752)	<0.001
AMH	1.062 (0.954–1.183)	0.271	1.173 (1.032–1.332)	0.014

Abbreviation: AMH, Anti-Müllerian Hormone; PCOS, Polycystic Ovary Syndrome; OR, Odds Ratio; CI, Confidence Interval; aOR, Adjusted Odds Ratio; BMI, Body Mass Index; CAMO, Composite Adverse Maternal Outcome; CAPO, Composite Adverse Perinatal Outcome.

## Data Availability

Due to hospital policies, patient data and study materials cannot be shared. However, the data are available from the corresponding author upon reasonable request.

## Abbreviations

The following abbreviations are used in this manuscript:

PCOS	Polycystic Ovary Syndrome
IVF	In Vitro Fertilization
AMH	Anti-Müllerian Hormone
GDM	Gestational Diabetes Mellitus
GDM A1	Diet-controlled GDM
GDM A2	Pharmacologically treated GDM
SGA	Small-for-Gestational-Age
LGA	Large-for-Gestational-Age
NICU	Neonatal Intensive Care Unit
CAMO	Composite Adverse Maternal Outcomes
CAPO	Composite Adverse Perinatal Outcomes
BMI	Body Mass Index
OGTT	Oral Glucose Tolerance Test
SD	Standard Deviation
IQR	Interquartile Range
OR	Odds Ratio
aOR	Adjusted Odds Ratio
CI	Confidence Interval
WHO	World Health Organization
NIH	National Institutes of Health
SPSS	Statistical Package for the Social Sciences
